# Antibacterial Compounds-Macrolactin Alters the Soil Bacterial Community and Abundance of the Gene Encoding PKS

**DOI:** 10.3389/fmicb.2016.01904

**Published:** 2016-11-29

**Authors:** Jun Yuan, Mengli Zhao, Rong Li, Qiwei Huang, Christopher Rensing, Waseem Raza, Qirong Shen

**Affiliations:** ^1^Jiangsu Provincial Key Lab of Organic Solid Waste Utilization and Jiangsu Collaborative Innovation Center for Organic Solid Waste Utilization – College of Resources and Environmental Sciences, Nanjing Agricultural UniversityNanjing, China; ^2^Fujian Provincial Key Laboratory of Soil Environmental Health and Regulation, College of Resources and Environment, Fujian Agriculture and Forestry UniversityFuzhou, China; ^3^J. Craig Venter InstituteLa Jolla, CA, USA

**Keywords:** macrolactin, soil bacterial community, PKS gene, MiSeq sequencing, microbial source antibiotics

## Abstract

Macrolactin produced by many soil microbes has been shown to be an efficient antibacterial agent against many bacterial pathogens. However, studies examining the effect of macrolactin on both the soil bacterial community and the intrinsic bacterial species that harbor genes responsible for the production of this antibiotic have not been conducted so far. In this study, a mixture of macrolactin was isolated from the liquid culture of *Bacillus amyloliquefaciens* NJN-6, and applied to the soil once a week for four weeks. 16S rRNA Illumina MiSeq sequencing showed that continuous application of macrolactin reduced the α-diversity of the soil bacterial community and thereby changed the relative abundance of microbes at both the phylum and genus level. The relative abundance of *Proteobacteria* and *Firmicutes* was significantly increased along with a significant decrease in the relative abundance of *Acidobacteria*. However, the application of macrolactins had an insignificant effect on the total numbers of bacteria. Further, the native gene responsible for the production of macrolactin, the gene encoding polyketide synthase was reduced in copy number after the application of macrolactin. The results of this study suggested that a bactericide from a microbial source could decrease the diversity of the soil bacterial community and change the bacterial community structure. Moreover, the populations of the intrinsic bacterial species which harbor genes responsible for macrolactin production were inhibited when the external source antibiotic was applied.

## Introduction

Soil harbors an aggregation of microorganisms containing millions of microbes per gram (Torsvik et al., 1990). These soil microbes play an important role in soil functions such as nutrition cycling, pollutant degradation and mass and energy flow (Bremner and Blackmer, 1978; Lewis et al., 1999; Barros and Feijóo, 2003). In addition, they influence both plant growth and productivity by supporting the production of antibiotics (Bentley et al., 2002; Newman and Cragg, 2012). Many reports found that soil microbes are a reservoir for the production of antibiotics, but only a small fraction has so far been discovered and characterized. Macrolide compounds are widely used antibiotics in clinics and display different biological activities including modulating inflammation (Kanoh and Rubin, 2010). Macrolactins are a large group of macrolide antibiotics with a 24-member lactone ring first discovered in a marine *Bacillus* (Gustafson et al., 1989). Macrolactin showed numerous properties such as inhibiting the proliferation of cancer cells, a protective effect on T-lymphocyte from an HIV infection due to the phosphatase inhibitory activity (Gustafson et al., 1989), anti-inflammatory activity towards colon epithelial cells, and the inhibition of cell division by the reduction of the H^+^-transporting two-sector ATPase, which is essential for the viability of bacterial cells (Romero-Tabarez et al., 2006; Zotchev et al., 2006; Park et al., 2014). Recently, its biocontrol activity to suppress many bacterial pathogens has been reported (Arguelles-Arias et al., 2009; Chen et al., 2009; Wang et al., 2012; Yuan et al., 2012a).

In the hospital or animal production facilities, the prescribed antibiotics are largely excreted by the recipient after administration (Halling-Sørensen et al., 2002; Thiele-Bruhn et al., 2004), resulting in high levels of antibiotic residues in animal manure or domestic waste water. Once macrolactins and their derivatives are applied along with manure, they tended to appear or even accumulate in the soil. In addition, it has increasingly been reported that macrolactins along with the microbes producing it, are being used to control soil-borne pathogen diseases in agricultural production (Han et al., 2005; Sopheareth et al., 2013; Yuan et al., 2013). Many biomimetic synthetic chemicals are being put into use for this purpose (Garson, 1993; Smith and Ott, 1996; Marino et al., 2002), thereby artificially increasing the concentration of macrolactin in soil. Macrolactin have an abroad-spectrum antibacterial activity against soil bacteria. There are many reports that antibiotics in soil can change the soil microbial community structure, increase the antibiotic resistance of soil microbes, and alter the original soil ecological function (Schmitt et al., 2004; Negreanu et al., 2012; Reichel et al., 2014; Udikovic-Kolic et al., 2014; Yamamura et al., 2014). However, the effects of macrolactin on the soil bacterial community and soil ecological functions are still only vague. Among the soil ecological functions, pathogen suppression is important for healthy plant growth, and antibiotic producing genes may be responses for soil pathogen suppression (Mendes et al., 2011). Macrolactin is synthesized by the polyketide synthase (PKS) (Schneider et al., 2007; Chen et al., 2009), and here the ketosynthase domain is most important for function (Schneider et al., 2007; Owen et al., 2013). In consideration of the antibacterial activity and the plant-induced elicitation of macrolactin in systemic resistance, the PKS gene may play a key role in the pathogen suppressive character of soil. However, there is only limited knowledge on the possible mechanisms of how the abundance of genes encoding PKS in soil could be altered when an excess of their respective gene products are suddenly supplied to the soil.

In our previous study, we isolated three macrolactin compounds (macrolactin A, 7-*O*-malonyl macrolactin A, and 7-*O*-succinyl macrolactin A) from the plant growth promoting rhizobacterium *Bacillus amyloliquefaciens* strain NJN-6 and these compounds displayed strong antibacterial activity, but very weak antifungal activity (Yuan et al., 2012a, 2014). In this study, we extracted a mixture of these three macrolactins and added them to the soil *in vitro*. Illumina MiSeq sequencing of the 16S rRNA gene region was performed to analyze changes in the soil bacterial community, and quantitative real-time PCR was used to evaluate variation in the abundance of the genes encoding PKS after exposure to macrolactins.

## Materials and Methods

### Isolation of Macrolactin Compounds

The antimicrobial compounds were isolated after the fermentation of strain NJN-6, which was isolated from the banana rhizosphere and identified as *B. amyloliquefaciens* by 16S rRNA sequencing (Yuan et al., 2012b).

For macrolactin isolation, strain NJN-6 was incubated in LB medium (10 g of tryptone, 5 g of yeast extract, and 10 g of NaCl per liter). For the production of antagonistic substances, the NJN-6 strain was grown in 1 L Erlenmeyer flasks with a 200 mL working volume, at 37°C and 170 rpm for 60 h. After wards the cell-free supernatant was collected by centrifugation at 12000*g* (4°C) for 10 min. Then an Amberlite XAD-16 (Alfa Aesar, a Johnson Matthey Company, Ward Hill, MA, USA) column (10 g) was used to absorb the active compounds. To remove the impurities, the column was first washed with 150 mL deionized water followed by 50 mL 30% methanol. The macrolactins were finally eluted with 100% methanol. The collected eluted fraction was concentrated by a rotary evaporator to remove the methanol. For further purification, the liquid was adjusted to pH 2.0 with 6 M HCl and stored at 4°C overnight. The precipitates were removed by centrifugation at 12000*g* (4°C) for 10 min, and the supernatants were re-adjusted to pH 7.0 with 6 M NaOH. The obtained solution was then freeze-dried into powder and re-solved in deionized water.

### Detection of Macrolactins by HPLC

HPLC was performed using a HPLC 1200 device (1200 series, Agilent, Santa Clara, CA, USA) to analyze macrolactins. For analysis, a 5 μL sample was injected into the HPLC column (Eclipse XDB-C18, 4.6 mm × 250 mm, 5 μm, Agilent, Santa Clara, CA, USA). The conditions were set up as described in our previous study (Yuan et al., 2012a). Briefly, the column temperature was maintained at 20°C throughout the analysis; the mobile phase was the solvent containing 60% A (0.1% (v/v) CH_3_COOH) and 40% B (CH_3_CN) at a flow rate of 0.6 mL/min; and an ultraviolet (UV) detector was used to detect peaks at 230 nm. There is no commercial standard sample for sale. The standard sample was obtained using high-speed counter-current chromatography (HSCCC) method reported previously (He et al., 2012, 2013). The concentration of macrolactins in the collected solution was quantified to be 38.6 mg/L.

### Soil Treatment with Macrolactins *In Vitro*

The soil was collected from an experimental site at “Wan Zhong” orchard (18°230′ N, 108° 440′ E), Le Dong County, Hainan Province, China. The experiment was performed *in vitro* with 15 g soil placed in a Petri dish with following treatments: (1) Control samples, the soil was amended with 1 ml sterile deionized water weekly; (2) treatment samples, the soil was amended with 1 ml sterile macrolactin solution weekly. Macrolactin solution or water was pipetted to soil lightly and mixed well by vortex. The whole treatment lasted for four weeks. All the Petri dishes were sealed with Parafilm and incubated at 30°C. Three replicates were performed for each treatment.

### Amplification and Sequencing of Bacterial 16S rRNA Genes

The DNA extracted (using the PowerSoil DNA Isolation Kit, MoBio Laboratories Inc., USA) from each soil sample (0.5 g) after 4 weeks inoculation served as the template for bacterial 16S rRNA gene sequence amplification. Three successive DNA extractions of each sample were pooled before performing polymerase chain reaction (to minimize the DNA extraction bias). The DNA quality was assessed according to the 260/280 nm and 260/230 nm absorbance ratios using a NanoDrop ND-2000 spectrophotometer (NanoDrop, ND2000, Thermo Scientific, 111 Wilmington, DE, USA). The concentration of extracted DNA was between 37 ng/μl and 61 ng/μl. The 520F (forward primer) and 802R (reverse primer) primer sets were used for V4 region amplification of the bacterial 16S rRNA gene. The primers used for final sequencing consisted of the appropriate Illumina adapter, pad linker, the gene-specific primer and a 6-nt barcode unique for each sample was attached to the reverse primer. The primers and the PCR condition are listed in Supplementary Table S1. PCR amplification was performed under the following conditions: the reaction mix (25 μl) contained 10 μmol of each primer (1 μl), 1 μl template DNA (20 ng/μl), 2.5 mmol of dNTPs (2 μl), 5× Q5 reaction buffer (5 μl) and 5× Q5 GC high enhancer (5 μl), 5 U/μl of Q5 polymerase (0.25 μl). After PCR amplification and agarose gel electrophoresis, bands were excised and purified using the MinElute PCR Purification Kit (Qiagen, Germany), separated by electrophoresis through a 1.5% agarose gel and purified from the gel using the Qiagen QIAquick Gel Extraction kit (Qiagen, Germany). The amplicons was subjected to unidirectional sequencing on the Illumina MiSeq sequencing platform of Personal Biotechnology Co., Ltd (Shanghai, China).

### 16S rRNA Sequencing Data Processing and Analysis

The sequencing data was processed with Mothur v.1.33.3 (Schloss et al., 2009) as described by Kozich et al. (2013). Make.contigs command was used to combine the two sets of reads due to the use of dual-index primers. Reads were screened using screen.seqs and reads that contained more than 0 ambiguous bases or were longer than 275 bp were removed from further analysis. Sequences were aligned to the SILVA bacterial database (Pruesse et al., 2007). Sequences that started and ended at the same position and had no more than 8 homopolymers were retained. Chimeras were detected using the Mothur implementation of UCHIME (Edgar et al., 2011). Detected chimeras were removed from the further downstream analysis. Sequences were classified using the Bayesian classifier against the Mothur compatible Ribosomal Database Project (RDP) training set version 10 (Wang et al., 2007). Taxonomic classification was based on RDP identification for each operational taxonomic unit (OTU). Sequences that were classified as Chloroplast, Mitochondria, unknown, or Eukaryota were removed from further analysis. An OTU-based approach was performed to calculate the richness and diversity using MOTHUR with an OTU cut-off of 0.03. The rarefaction curve was created to compare the relative levels of OTU richness across all soil samples. Richness indices of Chao1 and the abundance based on coverage estimator (ACE) were calculated to estimate the number of OTUs that were present in the sampling assemblage. The diversity within each individual sample was estimated using the nonparametric Shannon diversity index. The evenness of each individual sample was calculated based on the Shannon diversity index.

A multivariate data analysis was performed by using METAGENassist a web server tool (Arndt et al., 2012) that assigns probable microbial functions based on 16S rRNA data. In addition, principal component analysis (PCA) based on all taxa composition and relative abundance was conducted using the METAGENassist to better compare bacterial community similarities. SAS (ver. 9.3; SAS Institute) was used for statistical analyses. To determine statistical differences between treatments two-way ANOVA analysis with a Tukey post-hoc adjustment was used on log2 transformed data using the PROC MIXED function.

### Real-Time PCR for Genes Encoding PKS and 16S rRNA Genes

To generate external standard curves for real-time PCR assays, the microbial 16S rRNA and PKS were PCR-amplified from extracted DNA of *Bacillus amyloliquefaciens* NJN-6 with each pair of primers (listed in Supplementary Table S1), respectively. The PCR products were gel-purified using the Axygen gel extraction Kit (Axygen, Union City, CA, USA), cloned into pMD19-T vector and transformed into competent *Escherichia coli* Top 10 cells (Invitrogen, Carlsbad, CA, USA). The positive clones were selected and verified by re-amplification using the vector-specific primers T3 and T7, and further verified by DNA sequencing. Then, the insert clone of each target gene was selected to extract plasmid DNA with the Axygen Plasmid Miniprep Kit (Axygen, Union City, CA, USA). The plasmid DNA concentration was determined on a NanoDrop ND-2000 spectrophotometer (NanoDrop, Wilmington, DE, USA) and the copy numbers of each target gene was calculated directly from the concentration of the extracted plasmid DNA, respectively. Tenfold serial dilutions of a known copy number of the plasmid DNA were subjected to real-time PCR assay in triplicate to generate an external calibration curve. Amplification efficiencies with *R*^2^ values for 16S rRNA and PKs were 0.996 and 0.9994, respectively.

Real-time PCR was performed in biological triplicates and each involved three technical replicates with two negative controls on the Applied Biosystem 7500 Real-Time PCR System (ABI, USA) to enumerate the abundance of all the genes described above using SYBR Premix Ex Taq^TM^ (Tli RnaseH Plus) (TaKaRa Biotechnology Co., Ltd). The reaction mixture contained 10 μl SYBR Premix Ex Taq^TM^ (2×), 0.4 μl of each primer (10 pmol/μl), 0.4 μl ROX Reference Dye II, and 1 μl of template DNA (20 ng/μl) with a final volume of 20 μl. The primer sets and thermal conditions are listed in Supplementary Table S1. After the real-time PCR assay, the specificity of the amplification was verified by melting-curve analysis and agarose gel electrophoresis.

## Results

### Isolation of Macrolactins from NJN-6 Strain

To obtain macrolactins, resin absorbtion coupled with acid precipitation was used to remove the lipopeptide type impurities. A total of 50 ml concentrated solution was prepared from 8 L culture medium of the strain NJN-6. Three main peaks were detected in HPLC chromatography (Supplementary Figure S1), and then identified to be macrolactin A, 7-*O*-malonyl macrolactin A, and 7-*O*-succinyl macrolactin A when comparing the retention time with our previous results (Yuan et al., 2012a). The total concentration of macrolactins in the purified solution was 38.6 mg/L. The concentration of macrolactins in the soil (15 g) after application was calculated to be 2.57 μg/g for one time, and the total concentration after four applications was 10.29 μg/g.

### Effect of Macrolactin on the Soil Bacterial Community Composition

**FIGURE 1:**
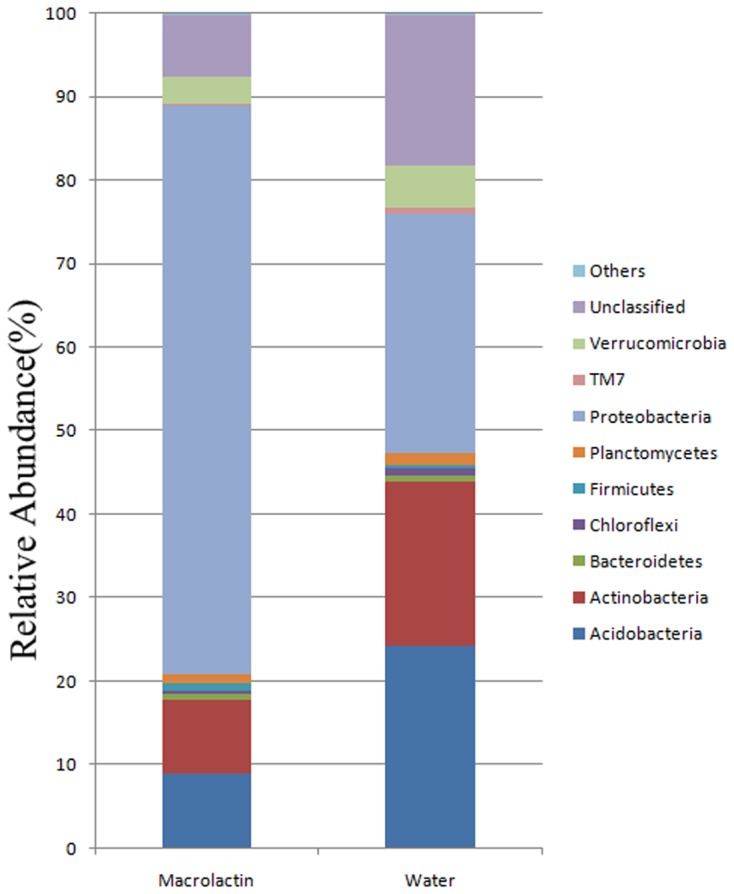
**Soil bacterial community composition based on 16S rRNA gene sequencing for macrolactin applied (macrolactin) and water applied (water) soil.** Community composition was shown at phylum level averaged for each treatment (*n* = 3).

A total of 232,559 sequences and 36,303-43,040 sequences per sample (mean = 38,760) were clustered to 18,808 OTUs using the average neighbor algorithm with a cut-off at 97% similarity. The raw sequence data have been deposited into the NCBI Sequence Read Archive (SRA) as study ID SRR4289338, SRR4292627, SRR4292629, SRR4292630, SRR4292631, and SRR4292670. Out of the total, 87.07% sequences could be classified at the phylum level, and 58.05% sequences could be classified to the genus level. When the OTUs were classified into phylotypes, the most abundant phyla were *Acidobacteria*, *Actinobacteria*, *Proteobacteria*, *Verrucomicrobia*, and unclassified groups, and these taxa accounted for more than 95% of the total sequences of both the macrolactin treatment and the control soil samples (**Figure 1**). In terms of phylum level changes after the addition of the macrolactins, the relative abundance of *Acidobacteria*, *Actinobacteria*, *Verrucomicrobia*, and unclassified groups was significantly decreased from 24.2%, 19.7%, 4.9%, and 18.0% to 8.9%, 8.8%, 3.3%, and 7.4%, respectively (**Figure 1**). *Protebacteria* the most abundant group was significantly increased from 28.8 to 68.3% when exposed to macrolactins (**Figure 1**). In addition, the *Firmicutes* were also found to be in more relative abundance in the macrolactin treated soil sample (from 0.47 to 0.94%).

We further analyzed at the genus level, and focused on the groups with a relative abundance higher than 1% in either one or both treatments. The results are listed in the **Table 1**. The relative abundance of genera Gp1, Gp2, Gp3, Gp13, *Marmoricola*, *Nocardioides*, *Phenylobacterium*, *Skermanella*, and *Spartobacteria_genera_incertae_sedis* was significantly higher in the control soil sample than those in the macrolactins treated soil sample; while the relative abundance of the genera *Burkholderia* and *Dyella* was significantly increased after macrolactins application. Surprising results were obtained for the genus *Rhodanobacter*, whose relative abundance was promoted by macrolactins from 3.25 to 33.56%.

**Table 1:** Frequency of the most abundant (>1%) classified bacterial genera (expressed as %) of all classified sequences within macrolactin treated soil and water control soil.

Phylum	Genus	Macrolactin	Control
Acidobacteria	Gp1	4.60 ± 0.59 b	11.64 ± 1.37 a
	Gp13	0.45 ± 0.14 b	2.03 ± 0.48 a
	Gp2	1.68 ± 0.27 b	6.74 ± 1.07 a
	Gp3	1.47 ± 0.20 b	3.05 ± 0.27 a
Actinobacteria	Marmoricola	0.07 ± 0.01 b	1.53 ± 0.22 a
	Nocardioides	0.06 ± 0.01 b	1.85 ± 0.17 a
Proteobacteria	Burkholderia	14.30 ± 4.33 a	1.94 ± 0.57 b
	Dyella	1.17 ± 0.64 a	0.06 ± 0.01 b
	Phenylobacterium	0.54 ± 0.13 b	1.23 ± 0.34 a
	Rhodanobacter	33.56 ± 7.02 a	3.25 ± 0.55 b
	Skermanella	0.93 ± 0.15 b	2.17 ± 0.40 a
Verrucomicrobia	Spartobacteria_genera_incertae_sedis	1.58 ± 0.14 b	2.80 ± 0.50 a

*The different letters “a” and “b” here used for the marker of statistically significant difference (p < 0.05), if there is significant difference between macrolactin treatment and water control, they are marked as “a” and “b” separately, if not, they are both marked as “a”. Data show a mean ±SD (n = 3)*.

### Changes of the Soil Bacterial Community Structure by Macrolactins

The rarefaction curve was firstly made at 97% similarity to reveal the increase of OTU numbers with the sequencing depth. The higher OTU number was observed in the control soil sample (water) compared to the macrolactins treated soil sample (macrolactin) (Supplementary Figure S2), which indicated that the macrolactins decreased the bacterial diversity when applied into the soil. Then we analyzed the α-diversity of the community structure to evaluate the effect of macrolactins on the soil bacterial community (**Table 2**). All the chosen parameters of α-diversity indices were significantly decreased by the macrolactins application, indicating the reduction of richness, diversity and evenness of the soil bacterial community.

**Table 2:** α-diversity indexes of soil microbial community in both macrolactin treated soil and water control soil evaluated by 16S rRNA gene sequencing.

	Chao	ACE	Shannoneven	Shannon
Macrolactin	10080 ± 734 b	19066 ± 938 b	0.57 ± 0.05 b	4.68 ± 0.44 b
Water	12087 ± 531 a	22161 ± 1178 a	0.73 ± 0.01 a	6.03 ± 0.08 a

*The different letters “a” and “b” here used for the marker of statistically significant difference (p < 0.05), if there is significant difference between macrolactin treatment and water control, they are marked as “a” and “b” separately, if not, they are both marked as “a”. Data show a mean ±SD (n = 3)*.

**FIGURE 2:**
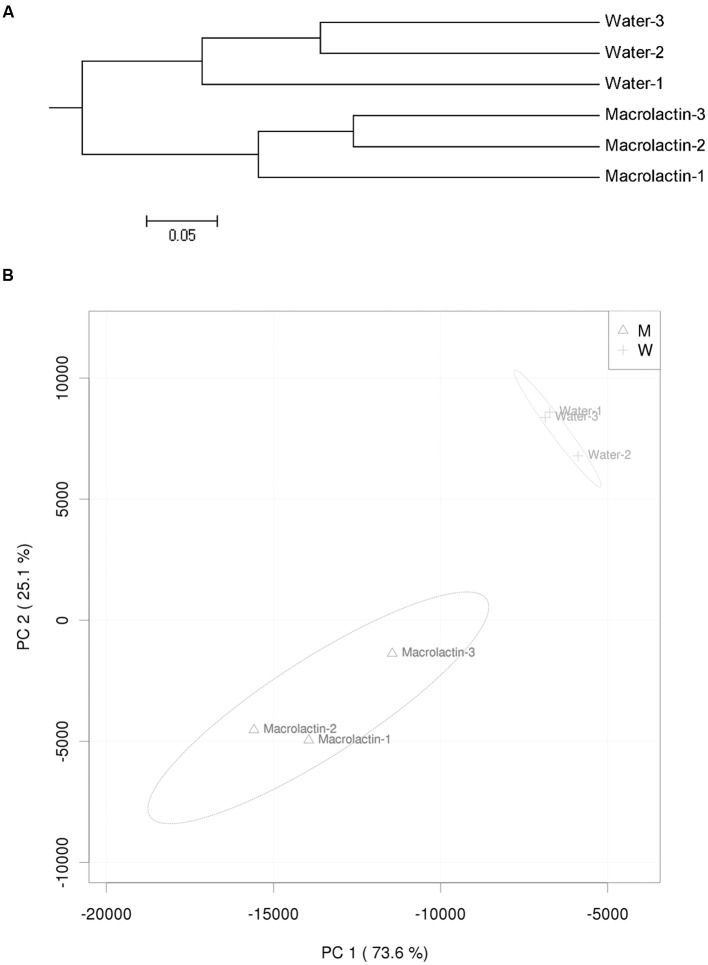
**(A)** Hierarchical cluster tree constructed based on the distance matrix that was calculated using the unweighted UniFrac algorithm for macrolactin treated soil and water control soil. **(B)** Principal component analysis (PCA) visualization of the pairwise community dissimilarity (Bray-Curtis index) of all tax levels in the microbial community analyzed by MiSeq sequencing. 95% confidence ellipses are shown around each treatment.

Hierarchical cluster analysis of the similarity of bacterial communities in this study confirmed that macrolactins application could significantly changed the bacterial community structure when compared to the control (**Figure 2A**). Furthermore, the PCA based on the abundance of all taxa showed the distance differences in the composition of the bacterial community in both the control and the macrolactins treated soil samples (**Figure 2B**).

### Influence of Macrolactins on the Abundance of 16S rRNA and PKS Gene

The quantitative real-time PCR data demonstrated that the copy number of the PKS gene was significantly reduced by the application of macrolactins when compared to the control (**Table 3**). However, the soil bacterial 16S rRNA gene abundance was decreased with no significant difference after the macrolactins application (**Table 3**).

**Table 3:** Real-time PCR quantification of 16S rRNA gene and PKS gene.

	16S rRNA log_10_(copy number)/g dry soil	PKS log_10_ (copy number)/g dry soil
Macrolactin	8.81 ± 0.22 a	7.32 ± 0.10 b
Water	9.01 ± 0.11 a	7.63 ± 0.21 a

*The copy number of genes in 1 g dry soil was estimated based on the results of real-time PCR. The different letters “a” and “b” here used for the marker of statistically significant difference (p < 0.05), if there is significant difference between macrolactin treatment and water control, they are marked as “a” and “b” separately, if not, they are both marked as “a”. Each sample was measured in triplicate, and data show a mean ±SD (n = 9)*.

### Macrolactins Influence the Potential Metabolic Function of Soil Microbial Community

**FIGURE 3:**
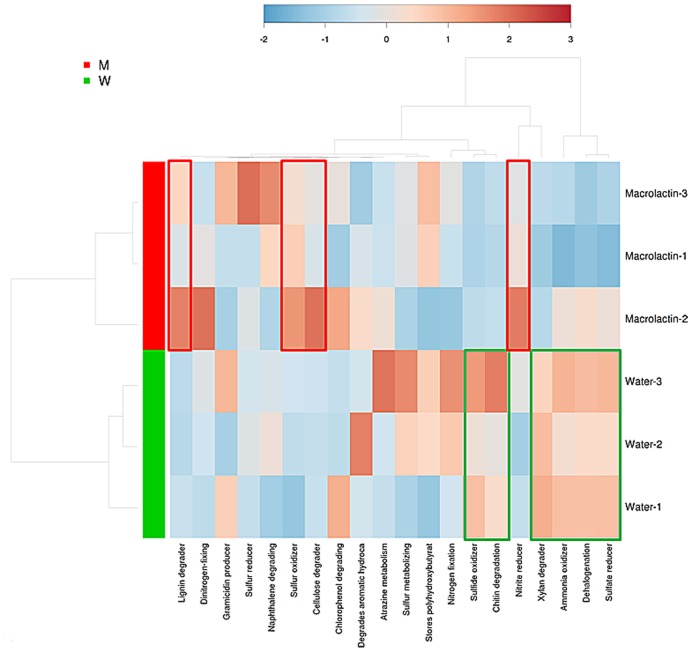
**Heatmap analysis of taxonomic to phenotypic mapping of the 16S rRNA sequenced genes between macrolactin treated soil and water control soil generated using METAGENassist**.

In order to predict the potential functions of the soil microbial community, we assigned the OTUs from taxonomic to metabolic function using the METAGENassist webserver tool. The heatmap analysis of the metabolic activities showed the differences between control and macrolactins treatment based on the Jensen-Shannon distance. The abundance of the genetic information encoding four bacterial metabolic pathways in soil (lignin degrader, nitrite reducer, sulfur oxidizer, and cellulose degrader) was elevated; while the abundance of other six metabolic pathways (chitin degradation, sulfide oxidizer, dehalogenation, xylan degrader, ammonia oxidizer, and sulfate reducer) was reduced by the application of macrolactins (**Figure 3**).

## Discussion

Macrolactins is a group of effective antimicrobial compounds, which are reported to show many clinically important biological activities, such as anti-cancer, anti- *Staphylococcus aureus*, and antiviral activities (Romero-Tabarez et al., 2006; Park et al., 2014). Recently, with the development of biocontrol applications for crop diseases, macrolactins were also found to be an effective biocontrol agent reported to antagonize many bacterial plant pathogens and played the main role in suppressing crop diseases (Jaruchoktaweechai et al., 2000; Romero-Tabarez et al., 2006; Chen et al., 2009). In our previous study, macrolactins were proven to be an antimicrobial agent to suppress the growth of *Ralstonia solanacearum* (Yuan et al., 2012a). In this study, many groups such as *Acidobacteria* and *Actinobacteria* were lower in abundance in the macrolactins treated soil sample compared to the control soil sample (**Figure 1**; **Table 1**), which again indicated a broad-spectrum antibacterial activity of macrolactins. A broad-spectrum antimicrobial activity is preferred in disease control as being able to inhibit several pathogens simultaneously. However, this broad-spectrum activity can also lead to major undesired consequences in the soil bacterial community because many non-target bacteria even beneficial bacteria could also be killed at the same time. In this study, the microbial antibiotic macrolactin displayed a similar disruptive effect on the microbial ecology as do agricultural chemicals (Monard et al., 2011). Some microbial groups were significantly reduced in relative abundance and other groups were significantly increased, while the total numbers of bacteria were not changed significantly as revealed by the qPCR analysis of 16S rRNA copy numbers after 4 weeks treatment. This might be due to sensitive groups being inhibited by macrolactins, while the resistant groups outgrew other groups due to fewer competitors.

When the effect of macrolactins on the soil bacterial community structure was taken into account, these compounds significantly reduced the bacterial diversity and richness (**Table 2**). The relative abundance of almost all the phyla except *Proteobacteria* and *Firmicutes*, was reduced (**Figure 1**), suggesting that the pre-existing balance of the soil bacterial community was disrupted by the addition of macrolactins. Similar reduction in the soil bacterial community and soil activity (enzyme activity) has been reported for the inputs of chemicals (Floch et al., 2011; Monard et al., 2011; Muñoz-Leoz et al., 2011; Jacobsen and Hjelmsø, 2014). At this point, there is no difference between the microbial antibiotic and chemically synthesized bactericides. The general taxonomic patterns largely differed between treatment and control soil sample regarding the abundances of major taxonomic groups (**Figure 1**). It was reported that the abundance of *Actinobacteria* and *Acidobacteria* depends on the soil moisture status and pH (Fierer et al., 2012; Barnard et al., 2013), therefore, the soil moisture status in this experiment was strictly controlled every week to rule out variations due to moisture differences. The macrolactins solution was adjusted to pH 7 and therefore there was no difference in pH between treatment and control soil samples. Although the *Actinobacteria* phylum was reported to have a high resistance to environmental stress due to its numerous members being Gram-positive with a high G+C content (Zviagintsev et al., 2007; Barnard et al., 2013). Based on our results, the conclusion that *Actinobacteria* and *Acidobacteria* were sensitive toward the macrolactin can be safely drawn.

On the other hand, the relative abundance of *Proteobacteria* (Beta-*proteobacteria* and Gamma-*proteobacteria*, Supplementary Table S2) and *Firmicutes* was significantly increased by the addition of macrolactins. These phyla have been reported to be more stable and resistant than other phyla when faced with changes of environmental factors (Barnard et al., 2013). Further, many species of *Firmicutes* are macrolactins producer such as *Bacillus*, that are resistant to macrolactins and elevate in relative abundance by their products (Yuan et al., 2012a). In the previous studies, the abundance of Beta-proteobacteria and Gamma-proteobacteriaalso showed a positive correlation with agricultural chemicals such as glyphosate, atrazine, permethrin and DDT as they were able to degrade these chemicals (Lew et al., 2013; Muturi et al., 2013). Beta-*proteobacteria* and Gamma-*proteobacteria* might be responsible for degradation of macrolactins in soil. The results of this study showed a decrease in the copy number of PKS synthase gene after the application of macrolactins. Other than Bacillus species, there are many soil microbes such as such as *Actinomyces* that contain gene encoding PKS synthase or similar genes (Ayuso-Sacido and Genilloud, 2005). In this study, *Actinobacteria* were significantly decreased in relative abundance by the application of macrolactins. This could be one reason that macrolactins applied to the soil reduced the relative amount of genotype known to encode PKS synthase.

At the genus level, among the 12 bacterial genera with over 1% relative abundance, the presence of 9 genera was reduced when exposed to macrolactins, which showed board-spectrum antibacterial activity of macrolactin on the bacteria. However, the relative abundance of the genera *Burkholderia*, *Dyella*, and *Rhodanobacter* was significantly increased (**Table 1**) which indicated that those groups were more resistant than others when exposed to macrolactin. These genera have been reported to play an important role in degradation of refractory pollutants especially agricultural chemical pesticides. *Burkholderia* displayed the ability to degrade organophosphorus pesticides in soil (Kikuchi et al., 2012; Pandey et al., 2012; Werren, 2012; Min et al., 2014). *Dyella* was a genus capable of degrading fenitrothion (Campisano et al., 2014; Itoh et al., 2014). The genus *Rhodanobacter* was able to remove benzo(α)pyrene and anilofos from the environment (Kanaly et al., 2002; Zhang et al., 2011).

The predicted soil bacterial functions, matched well with the soil bacterial community, were explored using METAGENassist which is a tool to predict the potential functions of the soil bacterial community, just as PICRUSt (Langille et al., 2013). It would have been better if the functional analysis had been done based on transcriptomic data. Current method monitors environmental DNA to predict microbial functions such as Geo-Chip, which could have given the relative abundance of distinct functional genes to predict the microbial functions in the habitat (He et al., 2007). For example, there were several species of *Rhodanobacter* found to be denitrifiers (van den Heuvel et al., 2010; Green et al., 2012), which might explain the significantly increased abundance of nitrite reducing metabolism (**Figure 3**). Here, we collected macrolactin compounds and evaluated their effects on the soil bacterial community. The results showed that the bacterial community diversity and the relative abundance of most taxa were decreased by the application of macrolactins. The increased relative abundance of *Proteobacteria* especially the taxa *Burkholderia*, *Dyella*, and *Rhodanobacter* might be due to higher resistance to macrolactins while *Acidobacteria* were sensitive to macrolactins in the soil. In addition, the abundance of genes encoding proteins responsible for antibiotic production (PKS gene) in soil could be reduced by the application of the microbial antibiotic (macrolactin). This study showed that microbial source antibiotics can change the bacterial community as agricultural chemicals do. However, it should be determined that how long it takes until macrolactins are totally degraded, and how long the effect on the bacterial community lasts to better manipulate soil microbial flora in the future.

## Author Contributions

JY and MZ: conducted all experiments, conceived the study, and wrote the paper; JY and RL: analyzed the data; QS, conceived the study, supervised the study, and wrote the paper; CR, WR, and QH: provided critical comments on the study, and helped write the paper.

## Conflict of Interest Statement

The authors declare that the research was conducted in the absence of any commercial or financial relationships that could be construed as a potential conflict of interest.
