# Supplementary material for: Antibacterial Compounds-Macrolactin Alters the Soil Bacterial Community and Abundance of the Gene Encoding PKS
Source: Front Microbiol. 2016 Nov 29;7:1904. doi: 10.3389/fmicb.2016.01904 (PMC5126139; doi:10.3389/fmicb.2016.01904)
Supplement: Supplementary file 1 [file Data_Sheet_1.DOCX]

Antibacterial compounds-macrolactin alters the soil bacterial community and abundance of the gene encoding PKS

Jun Yuan^a, 1^, MengliZhao^a, 1^, Rong Li^1^, Qiwei Huang^1^,Christopher Rensing^2^, Qirong Shen^1^^[[1]](#footnote-2)^*

^1^Jiangsu Provincial Key Lab of Organic Solid Waste Utilization; Nanjing Agricultural University, Nanjing, 210095, China.

^2^Institute of Urban Environment, Chinese Academy of Sciences, Xiamen, China

^a^ Both authors contributed equally to this paper

*Corresponding author: Dr. Qirong Shen, College of Resources and Environmental Sciences, Nanjing Agricultural University, 210095, Nanjing, Jiangsu Province, P. R. of China

E-mail address: shenqirong@njau.edu.cn, Tel: (86)02584396291; Fax: (86)02584396291

Running title: Macrolactin alters soil microbiome

Type of contribution: Research paper

Date of preparation: May 17, 2016

Number of text pages: 25

Number of tables & figures: 3 Tables and 4 Figures

Supplementary figure 1

HPLC chromatograph of the extracted macrolactin mixture extracted from fermentation of *Bacillus amyloliquefaciens* NJN-6. A, macrolactin A; B, 7-O-malonyl macrolactin A; C, 7-O-succinyl macrolactin A.


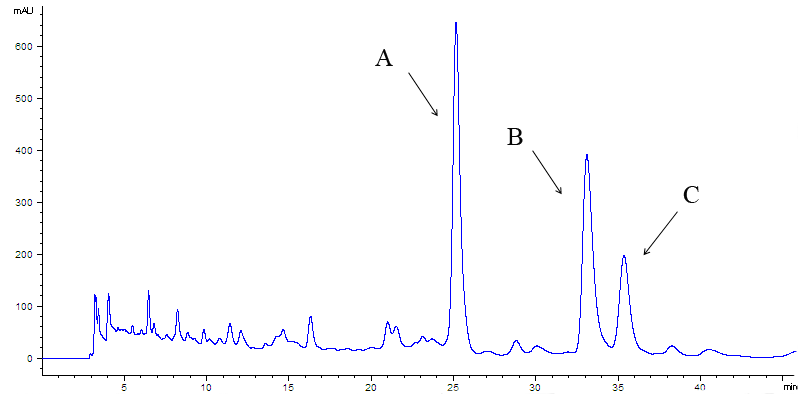


Supplementary figure 2

Rarefaction curves of bacterial communities based on observed OTUs at 3 % distance for individual samples. Error bars indicate 95 % confidence intervals


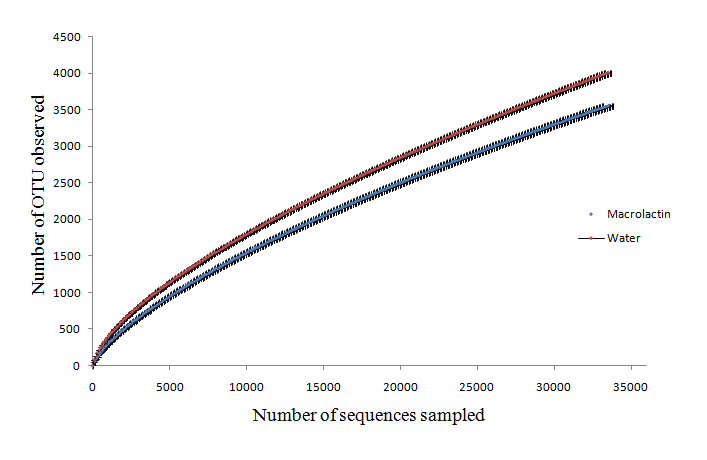


Supplementary Table 1. Primer sets and PCR conditions in this experiment.

| Target gene | Primer set | Sequence (5'-3')^a^ | Thermal profile | Reference |
| --- | --- | --- | --- | --- |
|  |  |  |  |  |
| 16S rRNA  (V4 region for sequencing) | 520F | A**Y**TGGG**YD**TAAAG**N**G | 30 s at 98 °C, followed by 27 cycles of 30 s at 98 °C, 30 s at 50 °C and 30 s at 72 °C, and a final elongation at 72 °C for 5 min | ([1](#_ENREF_1)) |
|  | 802R | TAC**NV**GGGTATCTAATCC |  |  |
| 16S rRNA  (for Q-PCR) | 347F | GGAGGCAGCAGT**RR**GGAAT | 30 s at 95 °C, followed by 40 cycles of 5 s at 95 °C, 34 s at 60 °C; melting curve program is 15 s at 95 °C, followed by 1 min at 60 °C, and continuous fluorescence measurement at 95 °C for 30 s,  and finally cooling of multiwell plate at 60 °C for 15s. | ([2](#_ENREF_2))  ([3](#_ENREF_3)) |
|  | 531R | CT**NY**GT**M**TTACCGCGGCTGC |  |  |
| PKS gene | KS2F | GC**I**ATGGA**Y**CC**I**CA**R**CA**RM**G**IV**T | 30 s at 95 °C, followed by 40 cycles of 5 s at 95 °C, 1 min at 55 °C, and 1 min at 72 °C; melting curve program is 15 s at 95 °C, followed by 1 min at 60 °C, and continuous fluorescence measurement at 95 °C for 30 s , and finally cooling of multiwell plate at 60 °C for 15s. | ([4](#_ENREF_4)) |
|  | KS2R | GT**I**CC**I**GT**I**CC**R**TG**IS**C**Y**TC**I**AC |  |  |

^a^ Boldface letters denote degenerate positions: R, A/G; S, G/C; Y, C/T; M, A/C; V, A/C/G; D, A/G/T; N, A/G/C/T. Rare bases: dI.

1. Claesson, M.J., et al., *Comparative analysis of pyrosequencing and a phylogenetic microarray for exploring microbial community structures in the human distal intestine.* PLoS One, 2009. **4**(8): p. e6669.

2. Kim, B.S., et al., *Rapid phylogenetic dissection of prokaryotic community structure in tidal flat using pyrosequencing.* J Microbiol, 2008. **46**(4): p. 357-63.

3. Nossa, C.W., et al., *Design of 16S rRNA gene primers for 454 pyrosequencing of the human foregut microbiome.* World J Gastroenterol, 2010. **16**(33): p. 4135-44.

4. Metsä-Ketelä, M., et al., *Molecular evolution of aromatic polyketides and comparative sequence analysis of polyketide ketosynthase and 16S ribosomal DNA genes from various streptomyces species.* Appl Environ Microbiol, 2002. **68**(9): p. 4472-9.

Supplementary table 2. The sequencing reads of order level which was significant different between treatments.

| Phylum | Order | Macrolatin | Control |
| --- | --- | --- | --- |
| **Acidobacteria** | Acidobacteria_Gp1 | 460±59 b | 1164±137 a |
|  | Acidobacteria_Gp13 | 45±14 b | 203±48 a |
|  | Acidobacteria_Gp2 | 168±27 b | 674±107 a |
| **Actinobacteria** | Actinobacteria | 882±124 b | 1970±365 a |
| **Bacteroidetes** | Sphingobacteria | 31±10 b | 56±7 a |
| **Firmicutes** | Clostridia | 41±24 a | 12±3 b |
| **Proteobacteria** | Alphaproteobacteria | 1293±555 b | 1974±21 a |
|  | Betaproteobacteria | 1638±474 a | 259±60 b |
|  | Deltaproteobacteria | 25±6 b | 39±7 a |
|  | Gammaproteobacteria | 3803±665 a | 511±105 b |
| **TM7** | TM7_class_incertae_sedis | 4±2 b | 72±24 a |
| **Verrucomicrobia** | Spartobacteria | 262±38 b | 392±65 a |
|  | Subdivision3 | 40±4 b | 74±13 a |

The different letters “a” and “b” here used for the marker of statistically significant difference (p<0.05), if there is significant difference between macrolactin treatment and water control, they are marked as “a” and “b” separately, if not, they are both marked as “a”. Data show a mean ± SD (n=3).

1. * To whom correspondence should be addressed: E-mail: [shenqirong@njau.edu.cn](mailto:shenqirong@njau.edu.cn) [↑](#footnote-ref-2)
